# The protein kinases family in fungi: adaptability, virulence and conservation between species

**DOI:** 10.3389/fmicb.2025.1630196

**Published:** 2025-08-15

**Authors:** Emanoelle La Santrer, Cláudia Barbosa Assunção, Thiago Miguelito Navarro de Camargo, Izabella Rodrigues, Sabrina Sidney Campolina, Edgar Lacerda de Aguiar, Thiago de Souza Rodrigues, Rachel Basques Caligiorne

**Affiliations:** ^1^Post-graduate Program in Medicine and Biomedicine, Faculdade de Saúde Santa Casa de Belo Horizonte, Belo Horizonte, Brazil; ^2^Post-graduate Program in Modelagem Matemática Computacional, Centro Federal de Educação Tecnológica de Minas Gerais – CEFET-MG, Belo Horizonte, Brazil

**Keywords:** kinases, GCN2, eIF2α, translational regulation, pathogenesis, virulence, stress response, amino acid deprivation

## Abstract

Protein Kinases (PKs) are a large family of enzymes that act as “molecular switches,” playing fundamental role in cellular signaling through protein phosphorylation. This process consists in transfer a phosphate group (*γ*-PO₄^2−^) from ATP (adenosine triphosphate) to specific residues in target proteins; thereby, controlling vital cellular processes, such as (i) cell proliferation and differentiation, (ii) response to environmental stimuli (stress, nutrients, hormones), (iii) metabolism, (iv) cell cycle and apoptosis, and (v) signal transduction. Among fungi, adaptability is intrinsically connected to their ability to thrive under extreme environmental stress, being morphological plasticity an example of this adaptability. While many of these adaptive responses are regulated by diverse signaling pathways involving different kinase families, as mitogen-activated protein kinase (MAPK) for example, this review places a special focus on the General Control Nonderepressible 2 kinase (GCN2), a highly conserved sensor of amino acid scarcity in many fungi, as well as the species *Cryptococcus neoformans*, *Candida albicans*, and *Aspergillus fumigatus*. Amino acid deprivation triggers the accumulation of uncharged tRNAs, which directly activate GCN2, and this activation leads to the phosphorylation of the eukaryotic initiation factor 2 alpha (*eIF2α*) at the serine in the position 51, initiating the Integrated Stress Response (ISR). Phosphorylated *eIF2α* suppresses global translation initiation while selectively enhancing the translation of stress-responsive genes, notably GCN4, which encodes a transcription factor that promotes amino acid biosynthesis and stress adaptation. In *Cryptococcus neoformans*, GCN2 emerges as the sole kinase responsible for *eIF2α* phosphorylation, a unique role in modulating translational responses to environmental and host-induced stressors. Previous studies have shown that the absence of GCN2 disrupts *eIF2α* phosphorylation, impairing stress responses and reducing pathogenicity, therefore being an important target for development of new generation antifungals. To better understand the mechanistic role of GCN2 and related kinases in amino acid sensing and stress response, we present a review based on studying the central role of kinases in fungal stress adaptation, discussing how the high conservation of their catalytic kinase domains makes them valuable as phylogenetic markers and therapeutic targets.

## 1 Introduction

Fungi occupy a distinct ecological and evolutionary niche characterized by a unique evolutionary lineage amongst eukaryotes (Webster and Weber, 2007; de Hoog et al., 2023; Gow et al., 2017). They inhabit soil and water, efficiently decomposing organic matter to recycle nutrients, colonizing plants or animals as pathogens, symbionts, or commensals, and their spores can be also found in airborne environments (de Hoog et al., 2023; Gow et al., 2017; Mcginnis and Tyring, 1996). As a general rule, fungi possess a rigid glucan and chitin-based cell wall, which confers structural integrity and resistance to environmental pressures, which is directly related with their evolution and adaptation (Mcginnis and Tyring, 1996; Brunke et al., 2016; Sun et al., 2020; Carbonero and Strobel, 2021; Kurokawa et al., 1998; Farrer and Fisher, 2017).

Adaptability is intrinsically connected to fungal survival and ability to thrive under extreme environmental stress as well as dimorphism is directly related to fungal adaptability (de Hoog et al., 2023; Seyedmousavi et al., 2014). Thus, dimorphic fungi are capable of transitioning between multicellular and unicellular morphs in response to changes in environmental conditions (Klein and Tebbets, 2007). The mycelial (multicellular) form is found in environmental conditions, consisting of hyphae (tubular elongated cells), while the yeast (unicellular) form is represented by oval cells (de Hoog et al., 2023; Ramírez-Sotelo et al., 2025). Fungal dimorphism is associated with changes in metabolism and cell wall remodeling and can be caused by a series of stimuli; however, temperature is often the primary regulator of dimorphism in fungi (Gauthier, 2017). In dimorphic pathogenic fungi, host temperatures (35–37°C) trigger the infectious yeast form, while cooler environmental temperatures (23–25°C) promote the mycelial form (Sacks et al., 1986). This morphological plasticity is regulated by complex signaling pathways, including kinase-mediated responses, which coordinate the cellular and metabolic changes necessary for fungal adaptation and pathogenicity (Pathan et al., 2019).

Protein Kinases (PKs) are a large family of enzymes that act as “molecular switches,” playing a fundamental role in cellular signaling through protein phosphorylation (Taylor and Kornev, 2011). This process consists of the transfer of a phosphate group (*γ*-PO₄^2−^) from ATP (adenosine triphosphate) to specific residues in target proteins and; thereby, controlling vital cellular processes, such as: (a) cell proliferation and differentiation, (b) response to environmental stimuli (stress, nutrients, hormones), (c) energy metabolism, (d) cell cycle and apoptosis, and (e) signal transduction (Taylor and Kornev, 2011; Gao et al., 2008; Wrabl and Grishin, 2001).

Considering the fungi kingdom, it is well known that kinases are commonly involved in three main biological mechanisms that directly impact the infection process: morphogenesis, virulence, and stress adaptation (Ramírez-Sotelo et al., 2025; Pathan et al., 2019). This adaptability is connected to thermotolerance, as the ability to survive at human body temperatures (37°C) is a prerequisite for pathogenicity (Mattoon et al., 2021; Money, 2024). For instance, black yeasts are a group of ascomycotan melanized fungi that perfectly exemplify these adaptive traits (de Hoog et al., 2023; Seyedmousavi et al., 2014; Assunção et al., 2020).

At the molecular level, adaptability is mediated by kinase signaling pathways, which regulate stress responses and morphological transitions (Cañete-Gibas and Wiederhold, 2018; Ene et al., 2014; Rauch et al., 2011). Our review analyzed and synthetized the current knowledge about the *eIF2α*-related kinases family, more specifically GCN2 kinases, which are regulators of amino acid biosynthesis and stress responses conserved across all fungal species, with potential molecular marker for studies on stress adaptability, virulence, target for new antifungal drugs and molecular evolution.

## 2 Kinases

Kinases are a class of highly conserved enzymes found across all the domains of life. They are activated by various regulatory signals, such as amino acid deprivation or oxidative stress (Rauch et al., 2011). These enzymes regulate cellular processes by catalyzing the transfer of phosphate groups to specific molecules in a process known as phosphorylation (Cohen, 2002). This post-translational modification occurs with a phosphate group (PO₄^3−^) transfer from a high-energy molecule to a specific target (Rauch et al., 2011; Hunter, 1991). Kinases selectively phosphorylate specific amino acid residues, such as serine (Ser), threonine (Thr), or tyrosine (Tyr) and are classified, considering their substrate specificity, in two types: Ser-Thr kinases, known for phosphorylating serine and threonine residues, and Thr kinases, that phosphorylate tyrosine residues. Also, some kinases are considered dual-specificity kinases for being capable of phosphorylating two substrates (Hunter, 1991; Wheeler and Yarden, 2015; Martin et al., 2010).

The family of enzymes catalyzing the phosphorylation of proteins, the protein kinases, represents a structurally diverse group of proteins (Taylor and Kornev, 2011; Martin et al., 2010; Ekhator et al., 2025; Yang, 2003; Defosse et al., 2015; Cheng et al., 2011). They are modular structures that differ widely in size, subunit structure, subcellular localization, mechanism of activation, and substrate specificity. Two general classes exist, those transferring phosphate to Ser or Thr and those transferring phosphate to Tyr (Hunter, 1991; Martin et al., 2010). According to an important study on evolution of kinases, Taylor and Kornev (2011) shows that despite this diversity, all eukaryotic protein kinases are evolutionarily related throughout a conserved catalytic core, indicating that they all share at least some common features of secondary and tertiary structures.

Ser-Thr and Tyr share the N-terminal ATP binding pocket, a conserved structural region and catalytic domain located at the N-terminal domain of the kinase, responsible for binding adenosine triphosphate (ATP) to amino acids (Hunter, 1991; Wheeler and Yarden, 2015; Martin et al., 2010). Thus, phosphorylation can be considered a molecular switch: it activates or inhibits enzyme function, affecting downstream signaling pathways, but it is not a permanent biochemical process, as it can be undone (Plattner and Bibb, 2011). There are regulatory mechanisms to ensure that phosphorylation is a reversible and controlled process (Cheng et al., 2011).

The cyclic AMP-dependent protein kinase has also been widely studied since many decades (Pir et al., 2025; Aryal and Watts, 2025; Scott, 1991; Xiong et al., 2025; Shabb, 2001; Jungmann and Russell, 1977). The cyclic AMP (cAMP) pathway represents a central signaling cascade with crucial functions in all organisms. In fungi, the cyclic AMP (cAMP) pathway plays a central role in regulating development, growth, differentiation, conidiation, germination and virulence of pathogenic species (Maeng et al., 2010; Ocampo et al., 2009; Zhao et al., 2006). Adenosine 3′,5′-phosphate (cyclic AMP) has been studied as an intracellular regulator in a wide variety of organisms. Studies of cyclic AMP function in fungi have concentrated on functions repressed by glucose as well as on diverse developmental and other specific responses reported to be influenced by cyclic AMP in animals (Sabina and Brown, 2009). Evidence has been obtained that links cyclic AMP to control of a variety of functions in fungi, including utilization of endogenous and exogenous carbon sources, conidiation (in *Neurospora crassa*), dimorphism in several fungi, the sexual process, and phototropism (in *Phycomyces*) (Davis, 1986; Judewicz et al., 1981; Wang et al., 2020). According some authors, there are some striking similarities between animal and fungal cyclic AMP-dependent protein kinases, which suggest strong evolutionary conservation of the properties of these enzymes (Müller et al., 2003; McDonough and Rodriguez, 2012).

The study of protein kinases in fungi has progressed significantly over the past decades, building a strong foundation for current models of stress response and morphogenesis. Table 1 lists some important studies on cyclic AMP-dependent protein kinases from different fungal species, which opened up perspectives for further studies.

**Table 1:** Studies reported on cyclic AMP-dependent protein kinases in different species of fungi kingdom.

Species	Reported kinase activity	Authors
*Arabidopsis thaliana*	Associated to reactive oxygen species (ROS)	Lokdarshi et al. (2022)
*Aspergillus fumigatus*	Associated to conidial germination, carbohydrate metabolism; virulence; morphology; sensitivity to oxidative damage.	Fuller et al. (2011); Zhao et al. (2006)
*Aspergillus nidulans*	Associated to glycogen synthase; microtubule-associated protein 2; synapsin; tubulin; gizzard myosin light chain; and casein.	Bartelt et al. (1988)
*Aspergillus niger*	Associated to morphogenesis; regulation of lipid biosynthesis as well as citric acid synthesis.	Štaudohar et al. (2002); Jernejc and Benčina (2003)
*Aspergillus parasiticus*	Associated to toxin synthesis, conidiation and aflatoxin synthesis.	Roze et al. (2004)
*Blastocladiella emersonii*	Associated to cyclic AMP-dependent protein kinase activity during sporulation and differentiation.	Juliani et al. (1979); Silverman (1978)
*Botrytis cinérea*	Associated to conidial germination, growth, and virulence.	Schumacher et al. (2008)
*Candida albicans*	Associated to morphogenesis, hyphal morphogenesis and virulence.	Gupta Roy and Datta (1986); Bhattacharya et al. (2023); Hossain et al. (2025);
*Candida auris*	Associated to Growth, Differentiation, Antifungal Drug Resistance, and Pathogenicity, with potential therapeutic strategies.	Kim et al. (2021)
*Candida tropicalis*	Associated to cAMP signaling pathway, morphogenesis regulation, and protein kinase A activity.	Kumar (2018); Nabeshima et al. (1977); Tanaka and Fukul (1977)
*Coprinus macrorhizus*	Associated to cAMP-dependent protein kinase activity and glycogen metabolism during mycelial development.	Uno and Ishikawa (1976)
*Cryptococcus neoformans*	Associated to Virulence and as potential targets for antifungical therapy.	Perlatti et al. (2020); Hu et al. (2007)
*Fusarium oxysporum*	Associated to vegetative growth, spore production, hyphal growth and mechanisms of fungal root pathogenesis.	Kim et al. (2011)
*Microsporum gypseum*	Associated to composition and regulation of phospholipid synthesis.	Dhillon et al. (2003); Khuller et al. (2000)
*Mucor circinelloides*	Associated to growth, differentiation, morphology, germination rates, cell volume, germ tube length, and asexual sporulation and branching.	Lübbehüsen et al. (2004); Ocampo et al. (2009)
*Mucor rouxii*	Polymeric structure characterization.	Moreno et al. (1977); Pastori et al. (1985)
*Neurospora crassa*	Associated to *eIF2α* phosphorylation, cpc-1 activation, general amino acid control (GAAC), nutrient stress adaptation.	Judewicz et al. (1981); Powers and Pall (1980); Štaudohar et al. (2002)
*Paracoccidioides lutzii*	Associated to phase transition, increasing during the mycelium to yeast transition.	Sestari et al. (2018)
*Penicillium oxalicum*	Associated to mycelial development, conidiation and the regulation of cellulase expression.	Sun et al. (2023)
*Saccharomyces cerevisiae*	Associated to phosphorylation-dependent regulation of translation, metabolic pathways (e.g., glucose fermentation, tricarboxylic acid cycle, pyruvate dehydrogenase and its bypass) and respiratory chain. Studies in evolutionary proteomics approach. GCN2 stimulates the expression of amino acid biosynthetic genes under conditions of amino acid starvation.	Hixson and Krebs (1980); Takai et al. (1974); Budovskaya et al. (2005); Ohlmeier et al. (2010); Wek et al. (1989); Gupta et al. (2025)
*Saccobolus platenses*	Polymeric structure characterization.	Silberstein et al. (1990)
*Trichoderma reesei*	Associated to light-modulated cellulase gene expression.	Schuster et al. (2012)
*Ustilago maydis*	Polymeric structure characterization.	Kerner and Passeron (1984)

As detailed in Table 1, certain kinases play critical roles in translational control under stress conditions. For instance, the GCN2 mediated phosphorylation of eIF2α, which leads to the selective translation of transcription factors like GCN4, which is a key adaptive mechanism in fungi that will be explored in detail in a subsequent section. To better appreciate the role of kinases such as GCN2 in contemporary fungal biology, we revisit early discoveries and methodological developments that shaped this field. According to (Uno and Ishikawa (1976), the activity of cyclic AMP-dependent protein kinase in mycelial extracts of *Coprinus cinerea* concurrently with decrease of glycogen content in mycelial cells. Also, Silverman (1978), as in other eukaryotic cells, cyclic AMP in *Blastocladiella* sp. regulates cellular activity by activation of cyclic AMP-dependent protein kinase. In 1979, Juliani et al. demonstrated that cyclic AMP-dependent protein kinase activity and cyclic AMP binding components are induced during the sporulation in *Blastocladiella emersonii*, corroborating with the previous study by Silverman (1978), with the same fungal species (Juliani et al., 1979).

The Ca2+-dependent protein kinase seems to be associated with membranous components, whereas cyclic GMP-dependent protein kinase appears to be related to certain subcellular organelle, such as nucleus. Suggestive evidence is available implying that the cyclic AMP-, cyclic GMP- and Ca2+-activated three sets of protein kinase systems may play each specific physiological role presumably owing to their own subcellular compartments (Davis, 1986; McDonough and Rodriguez, 2012).

A Ca2+/calmodulin (CaM)-dependent multifunctional protein kinase has been isolated from *Aspergillus nidulans* and purified to homogeneity. Unlike any CaM-dependent multifunctional protein kinase described previously, the native enzyme from *Aspergillus* behaves as a monomer (Jernejc and Benčina, 2003). According to the authors, the *Aspergillus* kinase catalyzes the Ca2+/CaM-dependent phosphorylation of known substrates of type II Ca2+/CaM-dependent protein kinases, including glycogen synthase, microtubule-associated protein 2, synapsin, tubulin, gizzard myosin light chain, and casein (Jernejc and Benčina, 2003).

Protein phosphatases counteract kinases by removing phosphate groups, restoring proteins to their unphosphorylated state, maintaining cellular homeostasis, and preventing aberrant signaling implicated in diseases (Narayanan et al., 2023). Protein kinases, in particular, are essential regulators of cell growth, differentiation, and apoptosis, acting during normal cellular function and disease states (Hunter, 1991; Donnelly et al., 2013). Thus, diverse studies have linked kinases as targets for therapeutic intervention, and numerous kinase inhibitors has been recently developed for cancer, systemic infections, and neurodegenerative diseases (Narayanan et al., 2023; Berkes et al., 2021; Piecyk et al., 2024).

In an opposite enzymatic process, protein kinase inhibitors are molecules that hinder the activity of protein kinases by binding to the kinase’s active site or inducing conformational changes, effectively blocking phosphorylation events and modulating cellular processes. Furthermore, kinase inhibitors have been investigated for their potential as antifungal agents (Berkes et al., 2021; Hinnebusch, 2011). An important study performed by Berkes et al. (2021), which identified the cancer therapeutic *dasatinib* as a potent inhibitor of *Histoplasma capsulatum* in its pathogenic yeast form, showing the potential of repurposing kinase inhibitors for antifungal therapy.

## 3 Scientometric analysis on kinase studies

To measure the scientific production related to kinases in fungi, we analyzed the research output using various scientometric indicators. These included publication trends in relevant journals, keyword analysis, and the distribution of publications by country, author, and institutional relevance, as well as collaboration networks. The initial search was conducted in the PubMed database using the search terms “kinases,” “fungi,” and “proteins” (van Eck and Waltman, 2010; Aria, 2017).

**Figure 1:**
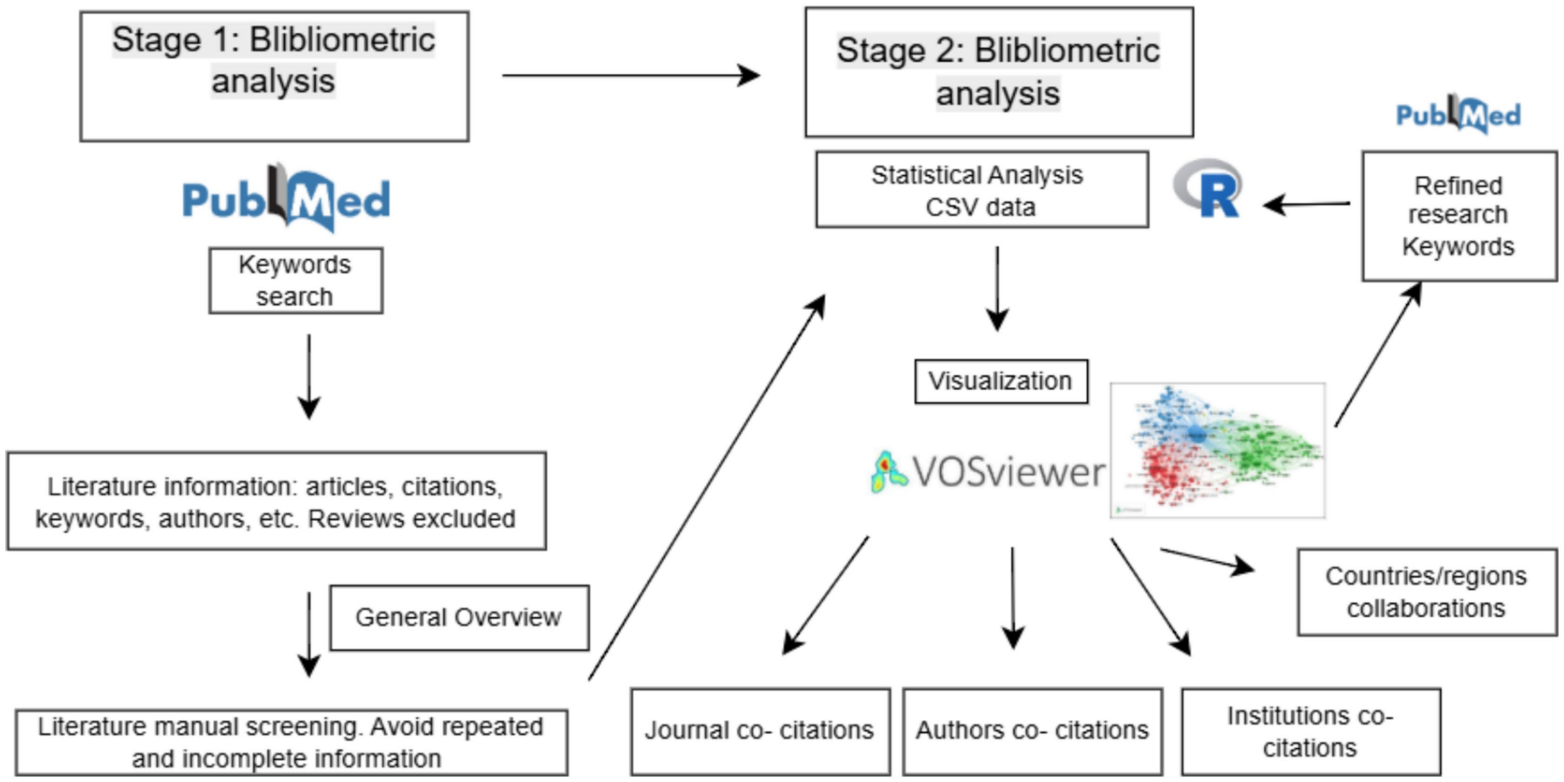
First, a broad overview of the kinase topic was conducted using the keywords “Fungi” and “Kinases.” Data from the 10,000 most relevant publications were manually screened to exclude reviews, duplicates, and publications with incomplete metadata. Resulting CSF generated data was analyzed using the R statistical program, and co-occurrence of major research topics was visualized with VOSviewer. Second, a refined analysis was performed using the keywords “dimorphic fungi” and “kinases.” The resulting dataset was manually screened according to the same criteria, and a second analysis was performed in R. VOSviewer was then used to visualize co-citation networks of countries, regions, journals, authors, and institutions.

The PubMed search yielded over 50,000 publications. Due to the processing capacity of the analysis software, the 10,000 most relevant articles with a specific focus on fungal kinases were selected for an initial overview. This subset was curated through manual screening to exclude reviews, duplicates, and studies with missing metadata (such as authorship, institutional affiliation, or publication year) or no abstract. The complete data selection and analysis workflow is illustrated in the scientometric analysis flowchart (Figure 1).

**Figure 2:**
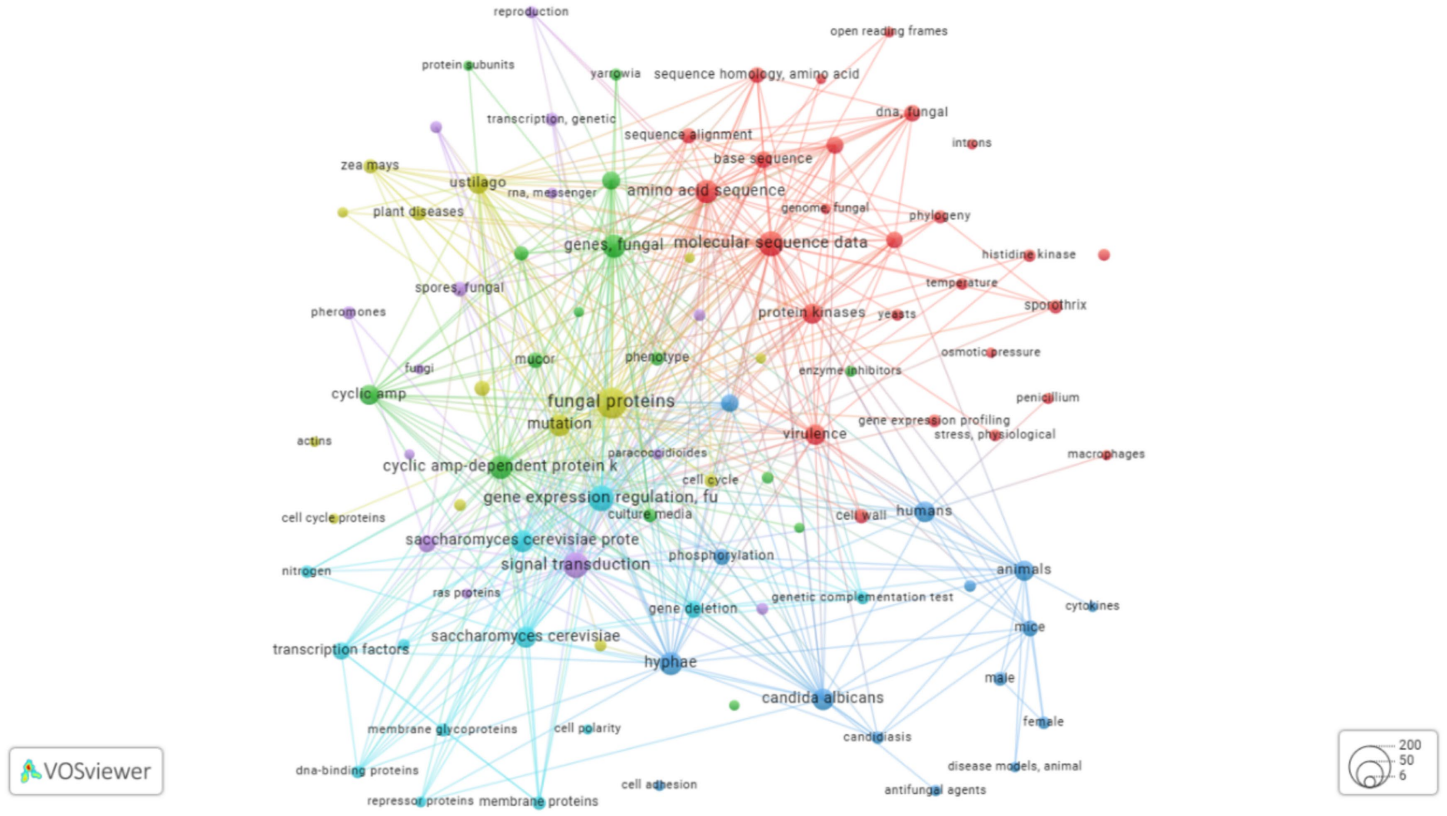
Keyword co-occurrence map generated using VOSviewer. The map is based on keywords extracted from approximately 270 articles on dimorphic fungal kinases retrieved from the PubMed database, published between 1982 and 2025. The analysis includes keywords that appeared at least five times. Colors denote thematic clusters of frequently co-occurring terms: the red cluster is associated with molecular data and phylogeny; the blue cluster relates to animal models and pathogenicity; and the green cluster represents cellular processes and fungal species. The size of each circle corresponds to the keyword’s frequency, with larger circles indicating terms that appeared more often.

Using this curated data, a CSF file was created and analyzed using R and visualized with VOSviewer (van Eck and Waltman, 2010; Aria, 2017). Keywords with at least 100 occurrences were included in this initial analysis (Lr, n.d.). Keyword analysis is critical as it indicates the focal areas of a research field and helps identify knowledge gaps. In this analysis, *Saccharomyces cerevisiae* was the most studied fungal species in the context of kinase proteins, followed by *Schizosaccharomyces pombe* (also called “fission yeast”), a common model organism in molecular and cell biology. *Candida albicans* was the third most studied species (Neiman et al., 1993). Regarding the biological activities of kinases, phosphorylation was the most extensively studied process. The analysis highlighted the importance of kinases in numerous cellular processes, including enzyme activation, gene expression regulation, protein binding, intracellular signaling, and transcription factor activity. Kinases are also involved in cell cycle progression, such as mitosis and DNA damage responses. Furthermore, studies on *Candida albicans* frequently associate kinase activity with virulence, demonstrating the functional versatility of these proteins (Figure 2).

To align with the focus of this review, a second, more targeted analysis was performed using the keywords “dimorphic fungal kinases.” Following the exclusion of reviews, duplicates, and articles with incomplete data, approximately 270 articles were selected from the PubMed database. Keywords appearing at least five times in this dataset are presented in Figure 2. In this focused analysis, *Candida albicans* remains the most prominent species, with *Sporothrix* and *Paracoccidioides* species also frequently appearing. Additionally, *Ustilago maydis*, a basidiomycete that causes corn smut, was identified (Olicón-Hernández et al., 2015). The term “Mucor” also appeared, which is associated with approximately 40 species of molds and dimorphic fungi, including both pathogenic and avirulent species (Lr, n.d.). This data demonstrates the importance of kinases in processes of morphological plasticity, such as dimorphism, and shows how molecular sequence data are crucial for uncovering kinase profiles and functions.

In addition to this keyword analysis, further scientometric analyses were conducted. The evolution of publications over the years, the most prominent authors and institutions, the most prolific journals, and existing collaboration networks were detailed. Graphical representations that complement these analyses are gathered in the Supplementary material of this study. The number of annual publications was evaluated. Publications on the subject began in the 1980s, with limited output until 1995, after which the number of publications doubled. The highest number of publications on kinases in dimorphic fungi was observed between 2000 and 2014. In 2015, there was a significant drop, followed by an increase and subsequent stabilization in 2018 and 2019 (Supplementary material).

A new drop in the number of publications was observed in 2020, likely because research institutions were focused on the COVID pandemic. The most productive institutions were also ranked, with the University of California leading (18 articles), followed by the Federal University of São Paulo (13) and the Institute of Microbiology (13). The University of Louisville (12) and the University of Buenos Aires (12) ranked fourth. The University of Murcia (11) and South China Agricultural University (11) followed, with Duke University Medical Center (10) and King’s College of London (9) completing the list (Supplementary material). Notably, two of the ranked institutions are in Latin America, likely reflecting the medical interest in dimorphic fungi in a region with a high incidence of fungal infections.

Evaluation of inter-institutional collaboration revealed no broad networks; however, collaborations between departments within Californian institutions were observed, which may contribute to the University of California’s leading rank (Supplementary material). The most prolific authors on this subject are Moreno, S. (10 articles), followed by Heitman, J. (9), and Ruiz-Herrera and Passeron, S. (seven articles each) (Supplementary material). Analysis of publication venues revealed that *Eukaryotic Cell* has published the highest number of papers (11), followed by *Molecular Microbiology* (10). *Fungal Genetics and Biology* and *FEMS Yeast Research* ranked third with six publications each (Supplementary material).

## 4 General Control Nonderepressible 2 (GCN2) kinase and its role in fungi

Amino acids are organic molecules fundamental to build proteins characterized by a central carbon atom bonded to an amino group (−NH3+), a carboxyl group (−COO–), a hydrogen atom and a variable side chain (R group), which determines the properties of each amino acid and consequently to protein structure and function (Donnelly et al., 2013; Douglas et al., 2024). In fungi, amino acids are fundamental for various biochemical processes, such as protein synthesis, enzyme production, and cell wall biosynthesis (Muhammad et al., 2024; Chadwick, 2023). They also act as precursors for important secondary metabolites, including pigments, toxins, and antimicrobial compounds, which are essential for fungal survival and pathogenicity (Ene et al., 2014). Amino acids represent an abundant source of nitrogen and carbon within the host and their process of acquisition by fungi interconnects sensory and uptake systems and downstream pathways (Maloy, 2013).

Amino acid starvation poses a significant challenge for fungi. Starvation is caused by an insufficient availability of amino acids due to environmental stressors (Ene et al., 2014; Chadwick, 2023; Garbe and Vylkova, 2019). Some environments are naturally poorer in nutrients, such as soil, air, or host tissue. Inside hosts, during pathogenic interaction, a sequester or degradation of essential amino acids can occur as an immunological mechanism, known as nutritional immunity, to starve the invading pathogen (Chadwick, 2023; Maloy, 2013; Garbe and Vylkova, 2019; Emery, 2012). Environmental stressors, including oxidative stress, temperature shifts, and osmotic pressure, can impair amino acid synthesis pathways or even completely deplete existing amino acid reserves (Maloy, 2013; Emery, 2012). Genetic mutations are also capable of impairing the endogenous amino acid production, leading to a starvation process even in environments rich in nutrients (Kim et al., 2011). Additionally, starvation induces autophagy, facilitating the recycling of intracellular components, including amino acids, to maintain essential metabolic processes (Maurin et al., 2022).

When faced with amino acid starvation, fungi activate stress response mechanisms to ensure survival and conserve energy, triggering a cascade of stress responses aimed at ensuring survival and adaptation (Knowles et al., 2021). The accumulation of uncharged tRNAs is a primary signal for amino acid scarcity, triggering the activation of the GCN2 kinase (Emery, 2012; McCarthy and Walsh, 2018). Recent studies reveal that fungi utilize multiple, interconnected pathways to detect and respond to amino acid scarcity (Emery, 2012; McCarthy and Walsh, 2018).

General Control Nonderepressible 2 kinase (GCN2) kinase activation by amino acid starvation also occurs in plants, mammals, and fungi through various regulation mechanisms, and it is quite conserved in those eukaryotes (Masson, 2019; Castilho et al., 2014; Maurin et al., 2005). Currently, GCN2 is identified as the sole kinase responsible for the phosphorylation of *eIF2α* (Leipheimer et al., 2019). In fungi, GCN2 phosphorylates *eIF2α eIF2α*, and, and it is composed of 1,649 amino acids, spanning from the N-terminal to the C-terminal region (Castilho et al., 2014). This phosphorylation event at serine 51 of *eIF2α* is a critical component of the Integrated Stress Response (ISR), allowing the fungus to modulate protein synthesis during environmental stresses (Masson, 2019; Castilho et al., 2014; Hinnebusch, 2005; Li et al., 2024).

The activation of GCN2 is triggered by the accumulation of uncharged tRNAs, leading to the phosphorylation of *eIF2α*, which causes a reduction in general translation initiation while selectively promoting the translation of stress-adaptive genes, more specifically GCN4 (Masson, 2019; Hinnebusch, 2005; Li et al., 2024). According to recent studies, in the absence of GCN2, *C. neoformans* exhibits impaired phosphorylation of *eIF2α*, resulting in reduced stress tolerance and diminished virulence (Leipheimer et al., 2019; Stovall et al., 2021).

In response to amino acid deprivation, yeast cells activate a signaling pathway involving GCN2-mediated phosphorylation of serine 51, which, in turn, triggers a two-pronged adaptation: global protein synthesis is down-regulated to preserve resources, while the translation of the GCN4 factor, which regulates the expression of a set of genes is preferentially promoted, stimulating the expression of genes responsible for amino acid biosynthesis (Hinnebusch, 2005; Li et al., 2024). While the fundamental architecture of GCN2 is preserved between animals and fungi, encompassing domains such as the RWD domain, pseudokinase domain (PKD), kinase domain (KD), histidyl-tRNA synthetase-like (HisRS-like) domain, and C-terminal domain (CTD), there are structural differences particularly in the CTD and dimerization interfaces since the GCN2 kinase in vertebrates has low sequence similarity when compared to the CTD of yeast GCN2, despite performing similar roles (Piecyk et al., 2024; Hamanaka et al., 2005).

**Figure 3:**
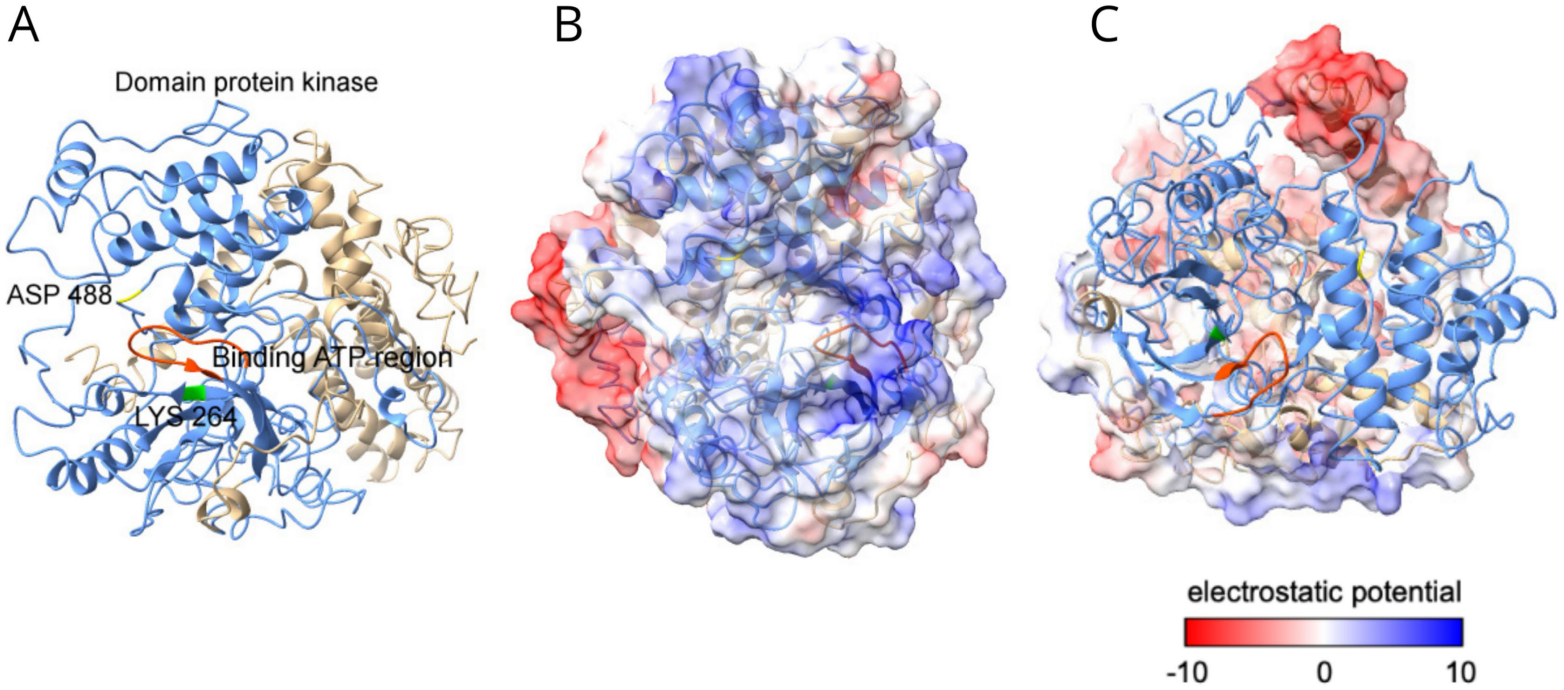
Structural and electrostatic analysis of the GCN2 protein from *C. carrioni*. **(A)** The overall domain architecture, illustrating the RWD, pseudokinase (PKD), catalytic kinase (PK), HisRS-like, and C-terminal domains that support its regulatory function. **(B)** A detailed view of the catalytic active site. Key residues lysine 264 (Lys264, shown in green) and aspartic acid 488 (Asp488, in yellow) are highlighted within the ATP-binding region (orange-red). These residues function as the proposed proton acceptor and donor. **(C)** Electrostatic surface potential of the kinase domain. Blue coloring represents positive charge (lower electron density), while red indicates negative charge.

As illustrated in Figure 3A, the GCN2 kinase is a multi-domain protein with five regions, a (1) RWD domain, (2) a pseudokinase domain (PKD), (3) Protein a Kinase (PK) domain, (4) a catalytic domain homologous to the histidyl-tRNA synthetase (HisRS), and a (5) C-terminal domain (Castilho et al., 2014). To better understand the structure of GCN2 protein, a predicted three-dimensional structure of the GCN2 protein from *C. carrioni* is presented in Figure 3 showing a detailed view of its functional architecture.

The RWD domain is a structural motif located in the N-terminal region of GCN2 kinase. It is named after its occurrence in RING finger-containing proteins, WD-repeat-containing proteins, and DEAD-like helicases, but in GCN2, it serves as a protein–protein interaction module, mediating interactions with GCN1 (Marton et al., 1993; Rakesh et al., 2017). It is where the assembly of the GCN2 kinase complex occurs (Castilho et al., 2014). It is highly conserved with approximately 110 amino acids, it adopts an α + *β* sandwich fold, and consists of a four-stranded antiparallel β-sheet and three α-helices. Masson (2019) describes the three-dimensional structure of each domain of the GCN2 enzyme. Represented by Protein Data Bank (PDB) codes, more specifically, the RWD domain structure exhibits an α + β sandwich fold, as observed in ID: 1UKX (Masson, 2019). Residues Glu123 and Glu136 in α-helix 3 are implicated in binding to GCN1, which facilitates the interaction between GCN2 and GCN1 (Rakesh et al., 2017; Nameki et al., 2004; He et al., 2014).

Additionally, a highly positively and negatively charged region is located near the RWD domain, including a pseudokinase domain (PKD), which resembles the Protein Kinase domain but lacks the residues necessary for enzymatic activity (Castilho et al., 2014). In spite of that fact, it acts as a structural scaffold, influencing the conformation and stability of the adjacent active Kinase domain (Stovall et al., 2021; Bou-Nader et al., 2024). Therefore, in order to modulate GCN2 activity, the PKD maintains the kinase in an inactive state under non-stress conditions and undergoes conformational changes upon stress signals, thus activating the functional Kinase domain (Masson, 2019; He et al., 2014).

A Protein Kinase domain that displays a typical kinase domain structure, represented by ID: 1ZYD, while the CTD is illustrated using the dimeric CTD structure ID: 4OTN. The crystallographic structure described by Padyana et al. (2005) suggests that the kinase domain maintains a “closed” conformation due to the rigidity of the hinge at the N- and C-terminal ends, bringing these regions closer together and partially obstructing access to the catalytic site. Furthermore, the kinase can undergo autophosphorylation at two threonine residues within the activation loop, leading to stabilization of the active state when the N and C lobes open. The formation of an intermolecular salt bridge between kinase domains facilitates this activation (Masson, 2019; Nameki et al., 2004; Padyana et al., 2005; Sattlegger et al., 2011). This formation needs to occur for the catalytic activity of GCN2, specifically the phosphorylation of *eIF2α*. It presents structural barriers that impede active conformation, and the PK domain forms a dimer during crystallization, with its protomers arranged in an antiparallel configuration (He et al., 2014). For the kinase to become active, it must undergo isomerization, shifting to a parallel orientation (He et al., 2014; Evelyn and Hinnebusch, 2000). Moreover, it can also adopt an active dimer configuration shared by the PKR and PERK2 kinase domains (Castilho et al., 2014).

Alongside the PK domain, the catalytic domain, repeat 2, of the Serine/Threonine kinase (STK)—central catalytic domain is similar to the homodimeric class II histidyl-tRNA synthetase (HisRS). In yeast cells, the GCN2 kinase is activated under amino acid starvation conditions through the binding of uncharged tRNA to its histidyl-tRNA synthetase-like (HisRS) domain (Bou-Nader et al., 2024; Wek et al., 1995). Another essential factor for GCN2 activation is the ribosome-binding and dimerization domain located in the C-terminal region (Ramirez et al., 1991). However, interactions between the HisRS and PK domains can hinder GCN2 activation, as this interaction reduces GCN2’s affinity for tRNAs (Bou-Nader et al., 2024). A kinase-like domain near the N-terminal region, along with the PK domain and the RWD region, functions as a regulatory domain. The RWD region mediates the binding of the GCN1—GCN20 complex, which interacts with translating ribosomes, facilitating the binding of uncharged tRNA to the HisRS domain and consequently activating the kinase (Castilho et al., 2014; Stovall et al., 2021; Padyana et al., 2005).

Lastly, the C-terminal domain which maintains GCN2 in an inactive conformation under non-stress conditions, when uncharged tRNA binding to the HisRS-like domain, conformational changes in the CTD relieve this inhibition and allow kinase activation, forming a bipartite tRNA-binding structure (Nameki et al., 2004; Sattlegger et al., 2011; Dong et al., 2000). Some studies implicate that the CTD negatively regulates kinase activity and that disrupting this interaction releases inhibition potentially enhancing GCN2 activation (Nameki et al., 2004; He et al., 2014; Dong et al., 2000). The CTD also facilitates ribosome association and stress response, and GCN2 must be physically tethered to the ribosome; therefore, involving CTD and associated regions for tRNA sensing and kinase activation (Liu et al., 2019).

## 5 Mechanisms of GCN2 activation and regulation by GCN1

GCN2 normally exists in an autoinhibited state, where its kinase activity is kept low to prevent unnecessary signaling. This autoinhibition is relieved by an allosteric activation mechanism that senses amino acid scarcity (Donnelly et al., 2013; Komar and Merrick, 2020). When amino acids are scarce, uncharged tRNAs accumulate in the cell, binding specifically to the HisRS-like domain, which is structurally similar to histidyl-tRNA synthetase but specialized for sensing these tRNAs rather than catalyzing tRNA charging (Bou-Nader et al., 2024). This binding event induces a conformational change that relieves GCN2’s autoinhibited state, allosterically activating its kinase domain (Komar and Merrick, 2020; Bou-Nader et al., 2024).

Once active, GCN2 phosphorylates the alpha subunit of the eukaryotic initiation factor 2 (eIF2α) at the serine-51 residue. This single phosphorylation event is the critical switch for cell modulation. The functional consequence is that phosphorylated eIF2α competitively inhibits its guanine nucleotide exchange factor, eIF2B (Sattlegger and Hinnebusch, 2005; Terenin et al., 2008; Zügel et al., 1999). Since eIF2B is required to recycle eIF2 from its inactive GDP-bound form to its active GTP-bound form, its inhibition leads to a sharp decrease in the available pool of active eIF2 (Sattlegger and Hinnebusch, 2005). This results in a potent suppression of global protein synthesis, conserving cellular resources during stress, while paradoxically enabling the selective translation of stress-response mRNAs, most notably that of the transcription factor GCN4 (Terenin et al., 2008; Zügel et al., 1999).

Normally, GCN4 translation is suppressed by upstream open reading frames (uORFs) in its mRNA. However, *eIF2α* phosphorylation alters ribosome scanning, bypassing these uORFs and successfully initiate translation at the main GCN4 start codon. GCN4, a transcription factor, then upregulates genes involved in amino acid biosynthesis and stress adaptation, facilitating cellular recovery (Hinnebusch, 2005; Li et al., 2024; Marton et al., 1993; Sattlegger et al., 2011).

Studies indicate that GCN2 must bind to GCN1, which selectively binds to GCN2 to regulate its biological activity. The kinase GCN1 is a cytoplasmic protein that plays a crucial role in helping cells respond to different stressors, and it also contains binding sites for GCN2 (Marton et al., 1993; Sattlegger et al., 2011). GCN1 comprises three regions, A, D, and E, which ensure that GCN1 can transmit signals to GCN2, enabling the cell to adapt to nutrient stress. Among GCN1 regions, region D is essential for GCN2 binding, mutations in this region are known to disrupt this interaction, preventing activation according to Masson (2019) and Hinnebusch (2005). While Region E weakens the connection, GCN2 overexpression can partially compensate as shown by Masson (2019) and Hinnebusch (2005). Region A, on the other hand, is the least critical and mainly helps stabilize the interaction rather than being directly responsible for binding (Hinnebusch, 2005; Sattlegger et al., 2011). GCN1’s middle region is homologous to the N-terminal HEAT repeat domain in EFF3 (Fungal translation elongation factor 3), which is essential for ribosome recycling and translational elongation, facilitating tRNA release from the E-site, where deacylated tRNA exits after peptide bond formation, and enhancing ribosome efficiency (Rakesh et al., 2017; Ranjan et al., 2021).

The interaction between GCN1 and GCN2, as well as their association with the ribosome, is required for GCN2 activation in both yeast and mammals (Gottfried et al., 2022). Hinnebusch (2005) identified an amino acid in GCN1’s GCN2-binding domain through protein interaction and functional analysis assay that is essential for this interaction, Arg2259, as its mutation disrupted the association but could be rescued by GCN2 overexpression (Hinnebusch, 2005). These findings confirm that the N-terminal domain of GCN2 must bind to the C-terminal region of GCN1 for optimal GCN2 function and amino acid regulation (Marton et al., 1993; Sattlegger et al., 2011; Gottfried et al., 2022).

The data support a model where GCN1, GCN20, and GCN2 form a ribosome-associated complex, with GCN1 aiding in the transfer of uncharged tRNA from the ribosomal A-site to GCN2’s tRNA-binding domain for kinase activation. Based on inferences drawn from several studies (Wek et al., 1989; Dong et al., 2000; Wek, 2018; Masson, 2019), Figure 4 summarizes the molecular mechanism of GCN2 activation and its role in fungal stress response.

**Figure 4:**
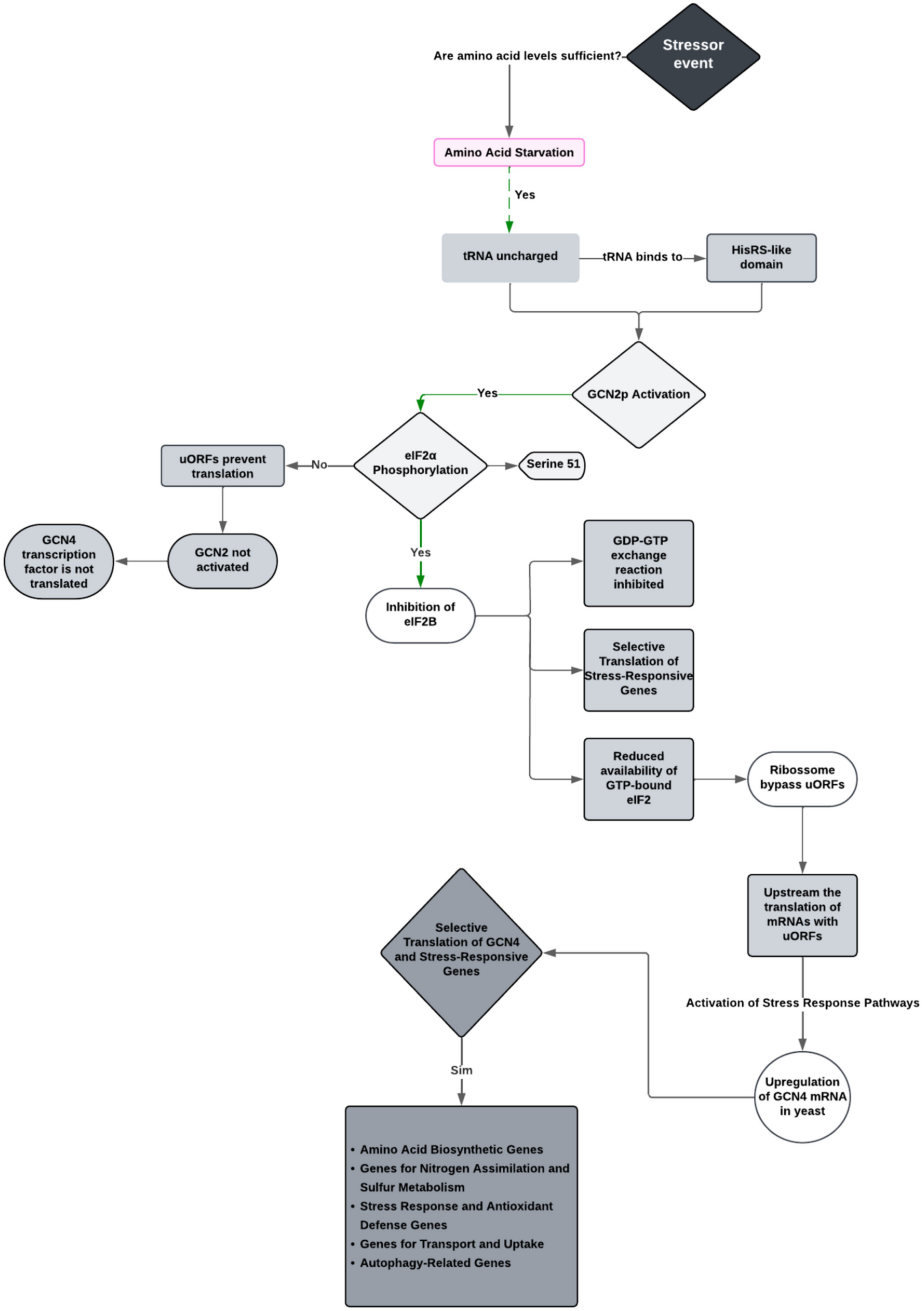
Integrated model of the GCN2-mediated stress response pathway in fungi. This diagram illustrates the cascade of molecular events triggered by amino acid starvation, including the activation of GCN2 by uncharged tRNA, phosphorylation of *eIF2α* at serine 51, inhibition of eIF2B, and the resulting selective translation of stress-responsive mRNAs such as GCN4. This pathway was constructed based on mechanistic insights from multiple authors (Hinnebusch, 2011; Masson, 2019; Castilho et al., 2014; Hinnebusch, 2005; Bou-Nader et al., 2024; Evelyn and Hinnebusch, 2000; Sattlegger and Hinnebusch, 2005; Teske et al., 2011).

## 6 Eukaryotic initiation factor 2 (eIF2)

Eukaryotic initiation factor 2 alpha, *eIF2α,* is an essential substrate within the Integrated Stress Response pathway (ISR), a conserved cellular signaling network that regulates protein synthesis in stressful conditions such as oxidative stress and amino acid deprivation (Donnelly et al., 2013; Pakos-Zebrucka et al., 2016). All this complex is considered a regulator of protein synthesis, modulating cellular responses to various stress conditions involved in phosphorylating the *eIF2α* subunit (Asada et al., 2025). The phosphorylation processes are necessary for the downregulation of global translation initiation, conserving resources and promoting stress adaptation allowing the selective translation of stress related protein, such as GCN4 (Wek, 2018). Eukaryotic initiation factors (*eIF*s) are necessary for translation initiation, and the *eIF*2 complex, including the regulatory subunit *eIF2α* is responsible for delivering initiator tRNA to the ribosome during the early stages of protein synthesis (Donnelly et al., 2013; Komar and Merrick, 2020; Burwick and Aktas, 2017).

There is an important distinction that deserves to be made: *eIF2α* is not a kinase since it does not add phosphate groups to other proteins. Instead, it is a substrate, phosphorylated by *eIF2α* related kinases at serine 51 (such as GCN2, PKR, PERK, HRI) (Donnelly et al., 2013; Komar and Merrick, 2020; Burwick and Aktas, 2017) The eIF2 Complex is a heterotrimeric GTP-binding protein composed of three subunits, *eIF2α*, eIF2*β*, and eIF2*γ*. The eIF2 participates in nearly all cytoplasmic mRNA translation events under normal cellular conditions (Kimball, 1999). *eIF2α* was initially identified as a factor capable of facilitating the binding of methionyl-initiator tRNA (Met-tRNAi) to the 40S ribosomal subunit in eukaryotic cells, in a process necessary for initiating protein synthesis through the formation of the 43S pre-initiation complex, which scans the mRNA to identify the start codon (Clemens, 1976).

Structurally, *eIF2* is a heterotrimeric complex consisting of three subunits: α (36 kDa), β (38 kDa), and γ (52 kDa) (Donnelly et al., 2013; Komar and Merrick, 2020; Kimball, 1999). Each subunit shows a unique functional role in translation regulation. The α-subunit contains a key regulatory serine residue at position 51, which is a major target for phosphorylation during stress responses, leading to translation inhibition (Kimball, 1999). This subunit has an important role as translational control in fungi and will be explored in depth in this review article. The β-subunit provides binding sites for the heterotetrametric guanine nucleotide exchange factor eIF2B, which converts protein synthesis eIF2 from a GDP-bound form to the active eIF2-GTP complex, facilitating the recycling of eIF2 between its active and inactive states, and eIF5, a GTPase-activating protein that enhances translation initiation (Castilho et al., 2014; Wek, 2018; Kimball, 1999). The γ-subunit, the largest of the three, binds guanine nucleotides (GTP and GDP) and Met-tRNAi, essential for forming the ternary complex required for ribosomal initiation (Donnelly et al., 2013; Komar and Merrick, 2020; Wek, 2018).

The *eIF2*-GTP-Met-tRNAi ternary is a prerequisite for the assembly of the 43S pre-initiation complex, including additional factors like *eIF1, eIFA*, and *eIF3.* This complex is responsible for scanning the mRNA and locating the right AUG start codon (Komar and Merrick, 2020). The translation initiation is highly dependent on eIF2, mediating the delivery of Met-tRNAi to the 40S ribosomal subunit. Ribosomes present two subunits for protein synthesis, in eukaryotic cells the 40s ribosomal subunit, also known as the small subunit is responsible for reading mRNA, ensuring that tRNAs are paired to their respective mRNA sequences to decode genetic information (Jennings et al., 2017). The Met-tRNAi delivery occurs through a ternary complex composed of *eIF2*, GTP, and Met-tRNAi. *eIF2* depends on its nucleotide-bound state to bind Met-tRNAi, and high affinity can be observed when *eIF2* is associated with GTP (Jennings et al., 2017; Kapp and Lorsch, 2004).

The *eIF5* facilitates the hydrolysis of *eIF2-*bound GTP by acting as a GTPase-activating protein and interacting with the 40S initiation complex, converting it to GDP. This conformational change results in the dissociation of *eIF2* from the ribosome in its GDP-bound form, along with *eIF5* (Unbehaun et al., 2004; Wortham and Proud, 2015). For *eIF2* to start again the translation initiation, its GDP must be replaced with GTP through a process mediated by *eIF2B*, a guanine nucleotide exchange factor composed of five subunits (α, β, γ, *δ*, and *ε*) (Castilho et al., 2014; Komar and Merrick, 2020; Kimball, 1999; Elsby et al., 2011). This phosphorylation event inhibits *eIF2B*, effectively halting the GDP-GTP exchange required for forming new ternary complexes and preventing the recycling of eIF2-GDP back to its active GTP-bound form (Terenin et al., 2008; Kapp and Lorsch, 2004; Unbehaun et al., 2004). Nevertheless, certain mRNAs can bypass *eIF2* dependence and continue to be translated through alternative initiation mechanisms such as internal ribosome entry sites (IRES) or re-initiation strategies (Evelyn and Hinnebusch, 2000; Ramirez et al., 1991). Selective translation of stress-response mRNA containing uORFs (upstream open reading frames), such as GCN4, continues (Hinnebusch, 2005; Li et al., 2024; Ramirez et al., 1991).

Four primary *eIF2α* kinases have been identified, each responding to distinct stress signals: Heme-Regulated Inhibitor Kinase (HRI), predominantly expressed in erythroid cells senses intracellular heme concentrations, exerting its functions through the dissociation of molecular chaperones such as Hsp90 and Hsc70. Heat shock proteins (Hsp) are a group of conserved chaperones that influence proteostasis and are responsible for activating the transcription of HSP genes under stress, protein folding, or proteasomal degradation (Zügel et al., 1999; Singh et al., 2024). It has been recently recognized that HRI is ubiquitously expressed and mediates *eIF2α* phosphorylation in various cell types exposed to multiple stressors (Lr, n.d.). Under heme-deficient conditions, heat shock, or heavy metal toxicity, HRI is activated, phosphorylating *eIF2α* to balance globin synthesis with available heme and; thereby, preventing the accumulation of unpaired globin chains (Donnelly et al., 2013; Burwick and Aktas, 2017; Girardin et al., 2021). This kinase responds to oxidative stress, as seen in its requirement for *eIF2α* phosphorylation and stress granule formation following sodium arsenite treatment, a potent inducer of oxidative damage (Burwick and Aktas, 2017; Girardin et al., 2021).

Double-Stranded RNA-activated Protein Kinase (PKR) is a vertebrate-specific kinase that serves as a key antiviral defense mechanism by recognizing viral double-stranded RNA (dsRNA), a byproduct of viral replication (Gal-Ben-Ari et al., 2019). Upon binding dsRNA, PKR undergoes dimerization and autophosphorylation via its C-terminal kinase domain, which is structurally preceded by N-terminal dsRNA-binding motifs (Gal-Ben-Ari et al., 2019). Once activated, PKR phosphorylates *eIF2α*, leading to the inhibition of guanine nucleotide exchange by eIF2B and subsequent suppression of cap-dependent translation initiation halting both viral and host protein synthesis (Gal-Ben-Ari et al., 2019; Yu et al., 2013). Beyond translational control, PKR can also induce apoptosis through *eIF2α*-independent mechanisms involving caspase pathway activation (Yu et al., 2013; Rothenburg et al., 2008). Dysregulation of PKR is associated with cancer, neurodegenerative diseases, and metabolic disorders (Gal-Ben-Ari et al., 2019; De La Cruz-Herrera et al., 2014; Dar et al., 2005). Given its central role in antiviral immunity, many viruses have evolved strategies to evade PKR activity, including direct inhibition, mislocalization, degradation, or interference with its RNA-binding capacity (Gal-Ben-Ari et al., 2019; Rothenburg et al., 2008; Dar et al., 2005).

Protein kinase RNA-like endoplasmic reticulum kinase (PERK) is a transmembrane protein residing in the ER capable or regulating cellular adaptation to ER stress. PERK is activated in response to the accumulation of misfolded proteins within the ER lumen, initiating the unfolded protein response (UPR) (Saito et al., 2011). In its inactive form, PERK is bound by the chaperone BiP (GRP78), but upon ER stress, BiP dissociates, allowing PERK dimerization and subsequent autophosphorylation at multiple serine/threonine residues, most notably at Thr980, which stabilizes its activation loop and αG helix for substrate interaction. Interestingly, PERK activation can also occur independently of misfolded proteins, possibly through fluctuations in ER luminal ATP or calcium levels sensed by BiP or associated co-chaperones (Saito et al., 2011; Cui et al., 2011). Once activated, PERK phosphorylates *eIF2α* and; thereby, inhibiting global cap-dependent translation and reducing protein load on the ER (Saito et al., 2011). This phosphorylation not only limits general protein synthesis but also enables selective translation of specific stress-responsive transcripts, such as ATF4, due to its unique upstream open reading frames (uORFs).

ATF4, in turn, regulates genes involved in redox balance, amino acid metabolism, and apoptosis, often converging on CHOP as a pro-apoptotic effector. PERK also directly phosphorylates the NRF2, disrupting its interaction with KEAP1 and facilitating its nuclear translocation to upregulate antioxidant genes. Notably, PERK signaling intersects with cell cycle regulation by repressing cyclin D1 translation, promoting G1 arrest to allow stress recovery (Saito et al., 2011; Cullinan and Diehl, 2004; Saptarshi et al., 2022). Recent findings further suggest PERK may act as a dual-specificity kinase, with tyrosine phosphorylation (Tyr615) contributing to its full activation. In cancer, PERK exhibits both tumor-promoting and tumor-suppressing functions depending on context (Teske et al., 2011; Saito et al., 2011; Saptarshi et al., 2022; Yan et al., 2002). Recent studies show PERK’s tumor-promoting activity in cancers such as ovarian carcinoma, where the persistent activation of the PERK-*eIF2α*-ATF4 signaling pathway promotes cancer cell survival, chemoresistance, and suppression of antitumor immunity via expansion and activation of myeloid-derived suppressor cells (Chen and Cubillos-Ruiz, 2021; He et al., 2024). PERK activation may also exhibit tumor-suppressive functions through induction of apoptosis by promoting expression of pro-apoptotic factors such as CHOP or by stimulating antitumor immune responses through mechanisms like paraptosis (Gibson et al., 2022).

Lastly, among the four known *eIF2α*-related kinases (PERK, PKR, HRI, and GCN2), GCN2 is the only kinase conserved and functionally relevant in fungi. While PERK and PKR are central to mammalian responses to ER stress and viral infection respectively, fungal cells rely exclusively on GCN2 to regulate translational control during nutrient deprivation, particularly amino acid starvation (Hamanaka et al., 2005; Li et al., 2023). This makes GCN2 the most relevant kinase that phosphorylates *eIF2α* in fungi, it is directly involved with fungal stress adaptation pathways and is currently the focus of this present study in order to better understand *eIF2α* regulation in fungi and dimorphic fungi (Masson, 2019).

Recent findings by Smirnova et al. (2025) suggest that natural compounds derived from fungi may influence stress response pathways involving *eIF2α*. In their study, the effects of *Wuling powder*, a preparation derived from the mycelium of *Xylaria nigripes,* was explored based on its capability of alleviating depressive-like behavior in mice (Li et al., 2016). Their findings showed that a modulation of endoplasmic reticulum (ER) stress pathways, involving the phosphorylated *eIF2α* and its downstream effector, activates the transcription factor 4 (ATF4) (Li et al., 2016). The direct involvement of GCN2 was not explicitly examined. Still, their findings imply a potential role for GCN2 in the observed stress response modulation, demonstrating how fungal metabolites might influence this signaling axis, potentially contributing to stress alleviation and neuroprotection (Li et al., 2016; Lin et al., 2013).

A recent phylogenetic and bioinformatic study focused on black yeasts has identified the hypothetical kinase gene in the *Herpotrichiellaceae* family (Assunção et al., 2024). According to this study, eIF2α is involved in stress response, suggesting that kinase proteins in black fungi could be a potential target for antifungal therapy, based on the disruption of translation regulation that negatively affects their survival under host-induced stress (Assunção et al., 2024). More recent research has shown that even in the absence of stress, GCN2 is capable of preventing excessive ribosome biogenesis and mRNA translation, maintaining proteome stability (Román-Trufero et al., 2025). Another study focused on GCN2 signaling in mammals has shown that GCN2 influences lipid homeostasis by targeting key transcriptional regulators of lipogenesis, thereby linking the integrated stress response to the control of cellular lipid metabolism, which leads to the reinforcement of GCN2’s function in regulating cellular adaptation (Guo and Cavener, 2007).

On the other hand, in specific pathological states, the GCN2-mediated stress response can be detrimental. In the context of diabetic cardiomyopathy, reducing GCN2 levels has been shown to exert a cardioprotective effect (Yuan et al., 2022; Dabravolski et al., 2021). The mechanism for this protection involves the inhibition of the canonical eIF2α-ATF4-CHOP signaling pathway, leading to a decrease in oxidative stress, apoptosis, and harmful lipid accumulation in cardiac cells (Dabravolski et al., 2021). These seemingly contradictory findings show the context-dependent and tissue-specific role of GCN2 in metabolic regulation, where its activation can be either protective or detrimental depending on the physiological state.

Another study shows that GCN2 activation during leucine deprivation suppresses hepatic lipogenesis and promotes fatty acid oxidation, helping to prevent lipid accumulation in the liver (Kurokawa et al., 1998). GCN2-deficient mice displayed increased liver triglycerides and impaired expression of genes involved in *β*-oxidation (Kurokawa et al., 1998). This could signify that GCN2 is a promising therapeutic target to mitigate cardiac damage in diabetic patients, with the potential to counteract the cardiotoxic side effects of certain drugs (Yuan et al., 2022; Dabravolski et al., 2021). Which would fit in GCN2’s regulatory function.

Also, further studies show that in *Pestalotiopsis microspora*, an endophytic fungus able to digest polyurethane-based plastic materials, GCN2 deletion mutants impaired conidiation, disrupting secondary metabolism, and causing defective cell wall integrity, which indicates GCN2’s potential role in typical morphological development and stress responses in filamentous fungi (Jin et al., 2022). Regarding GCN2 inhibition research, studies have shown promising therapeutic potential, particularly in oncology and neurodegenerative disorders. A study details the potential of a GCN2 modulator to overcome drug resistance, a major clinical challenge, based on preclinical efficacy of HC-7366, a GCN2 activator, in AML models, which induces significant anti-tumor effects both as a standalone therapy and in combination with the standard-of-care drug (Tameire et al., 2023).

Drug resistance significantly impacts treatment options, which presents a challenge, especially when treatment options are limited. In view of this cancer research has been investigating novel therapeutical options, in special, an AACR abstract introduces CRD-799, a novel oral inhibitor that targets not only GCN2 but also two other related stress-response kinases, HRI and PERK. The study shows that in multiple myeloma, a cancer of plasma cells, combining CRD-799 with proteasome inhibitors (a standard treatment) can overcome drug resistance (Inoue et al., 2024). In the same line, another study investigates the preclinical effectiveness of a new GCN2 inhibitor, KAS-1155 on rhabdomyosarcoma, a rare cancer affecting soft tissue. By inhibiting GCN2, KAS-1155 could disrupt the cancer cells’ ability to adapt to stress, leading to cell death and opening the pathway for more viable therapeutic strategies (Kang et al., 2024).

Expanding the therapeutic potential of GCN2 inhibitors beyond oncology, this study investigates their role in the neurodegenerative disease ALS. The research found that inhibiting GCN2 could reduce the toxic aggregation of mutant SOD1 protein, a crucial pathological element in some forms of ALS. In a mouse model of the disease, GCN2 inhibition delayed disease progression, suggesting that this mechanism could be a promising neuroprotective strategy (Ortiz et al., 2024). The preclinical success of these GCN2 inhibitors in complex mammalian disease models provides a strong rationale for exploring their efficacy as antifungal agents.

It is important to consider the effects of known pharmacological inhibitors when it comes to the therapeutic relevance of GCN2, studies have shown that ATP-competitive kinase inhibitors can inadvertently activate GCN2 by stabilizing its active conformation, rather than inhibiting it (Tang et al., 2022; Kato et al., 2020; Nakamura et al., 2018). This paradoxical activation suggests that drug interactions with GCN2 are more complex than previously assumed and could influence outcomes in diseases where GCN2 plays a regulatory role (Kato et al., 2020). Selective GCN2 inhibitors such as GCN2iB have demonstrated efficacy in modulating stress responses and suppressing tumor growth in preclinical models (Kato et al., 2020; Nakamura et al., 2018). It is possible to conclude that inhibiting GCN2 in fungal pathogens could offer therapeutic benefits in diverse fields. Dissecting the specific roles of this conserved pathway in pathogenic fungi remains a crucial step toward validating GCN2 as a robust and safe antifungal drug target.

## 7 Conclusion

- This review provides a detailed analysis of the eIF2α kinase GCN2 and its role in fungal amino acid sensing and stress response. We focus on the molecular mechanisms of GCN2 function and it signaling pathways, while also surveying the latest research trends in the field. This growing interest points toward underexplored fungi and mechanisms that could drive future discoveries, setting a path for advancing the field of microbiology.
- Adaptive capacity directly impacts fungal survival, development, and pathogenicity. As the only conserved and active eIF2α-related kinase in fungi, GCN2 has a strong potential to become a universal stress response marker. Such a marker can be particularly valuable for genetically diverse pathogenic fungi, where identifying conserved pathways is often a challenge.
- Kinase GCN2’s role and conservation in eukaryotes have shown its potential as a target for novel antifungal therapies, especially as translational control gains recognition for pathogen management. Beyond its role in sensing amino acid deprivation, recent studies indicate that GCN2 also participates in other cellular processes, such as lipid homeostasis, linking translational control to more ample metabolic regulation. This has spurred the investigation of GCN2 inhibitors, many initially developed for oncology, as potential antifungal agents.
- A critical consideration for GCN2 as a therapeutic target is its feasibility, given the existence of a human homolog, EIF2AK4. While the catalytic kinase domain is highly conserved across eukaryotes, significant structural divergences exist between fungal and human GCN2, particularly in the regulatory domains. As this review notes, regions such as the C-terminal domain (CTD) and dimerization interfaces show low sequence similarity when comparing fungal and vertebrate kinases. These differences in the non-catalytic domains, which are crucial for controlling kinase activation, offer a promising strategy for the rational design of fungus-specific inhibitors. By developing molecules that target these less-conserved regulatory regions instead of the active site, it is feasible to achieve high specificity, thereby minimizing off-target effects on the human homolog.
- Considering the rising challenge of fungal resistance, a deep understanding of how fungi adapt at the molecular level is more critical than ever. We believe this framework will guide future research and encourage collaboration across microbiology, genomics, structural biology, and drug discovery, ultimately supporting efforts to combat fungal diseases more effectively.
