# Supplementary material for: The protein kinases family in fungi: adaptability, virulence and conservation between species
Source: Front Microbiol. 2025 Aug 15;16:1630196. doi: 10.3389/fmicb.2025.1630196 (PMC12394161; doi:10.3389/fmicb.2025.1630196)
Supplement: Supplementary file 1 [file Data_Sheet_1.docx]

**SUPPLEMENTARY MATERIAL –**

**FIGURES OF SCIENTOMETRIC STUDIES**


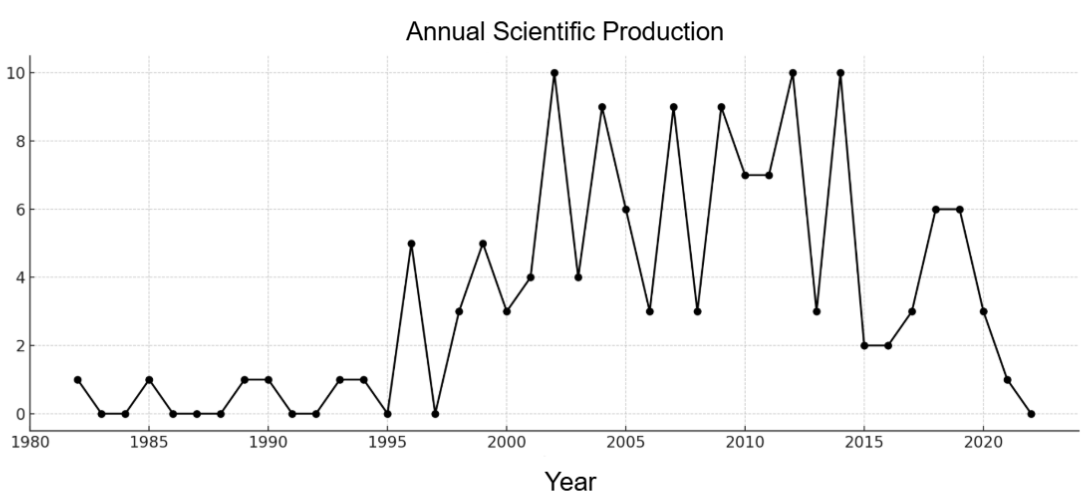


Figure 1. Annual distribution of scientific publications related to "dimorphic fungal kinases", based on data retrieved from the PubMed database (~270 articles) between 1982 and 2025. Data were analyzed using the Bibliometrix R-package. The increasing publication trend over the last two decades highlights scientific interest in fungal kinases as potential therapeutic targets.


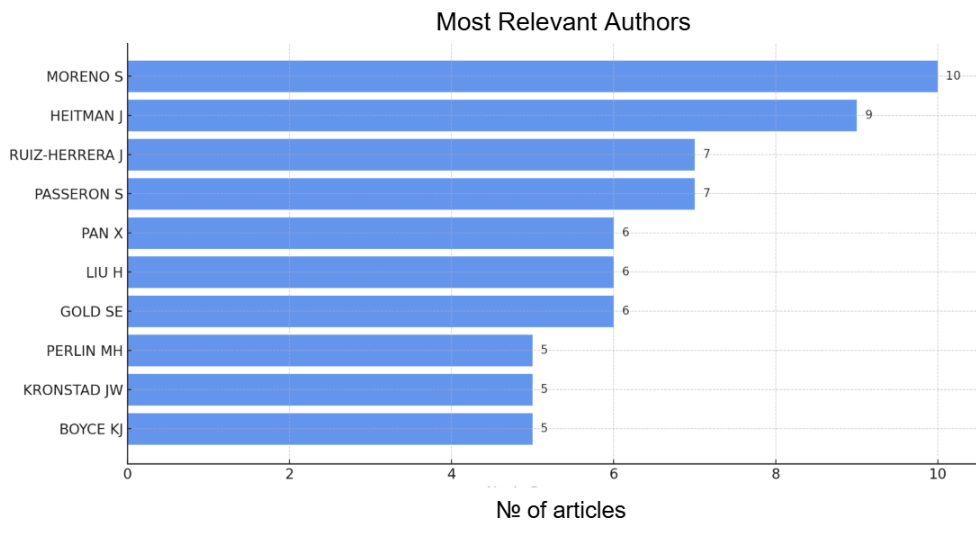


Figure 2. Author productivity identified through scientometric analysis using the Bibliometrix R-package based on PubMed database (~270 articles) between 1982 and 2025. It was based on publications number and collaboration networks. These findings highlight researchers with significant influence and visibility in fungal kinase research.


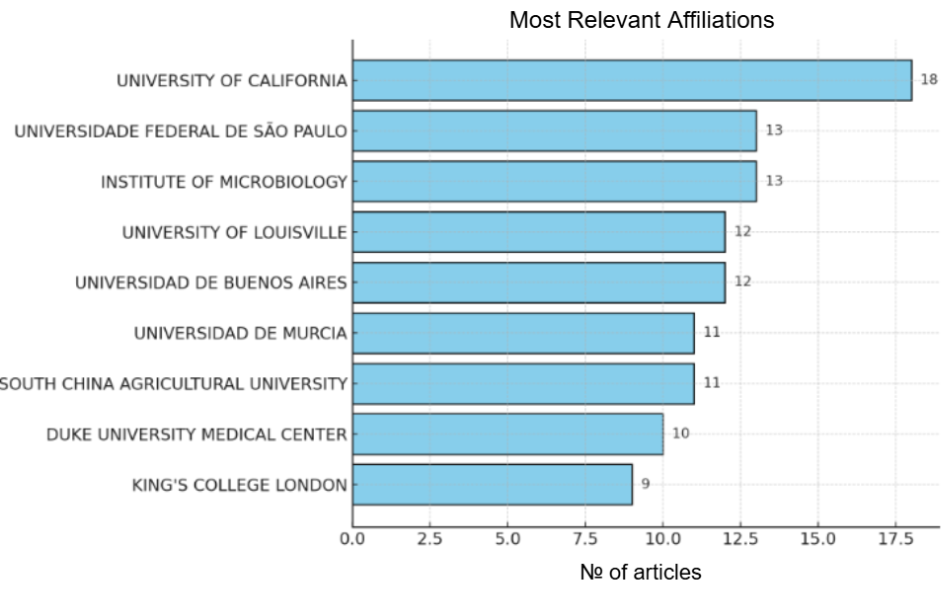


Figure 3. Academic and research centers actively involved in dimorphic fungal kinases. The analysis were performed using the Bibliometrix R-package based on PubMed database (~270 articles) between 1982 and 2025.


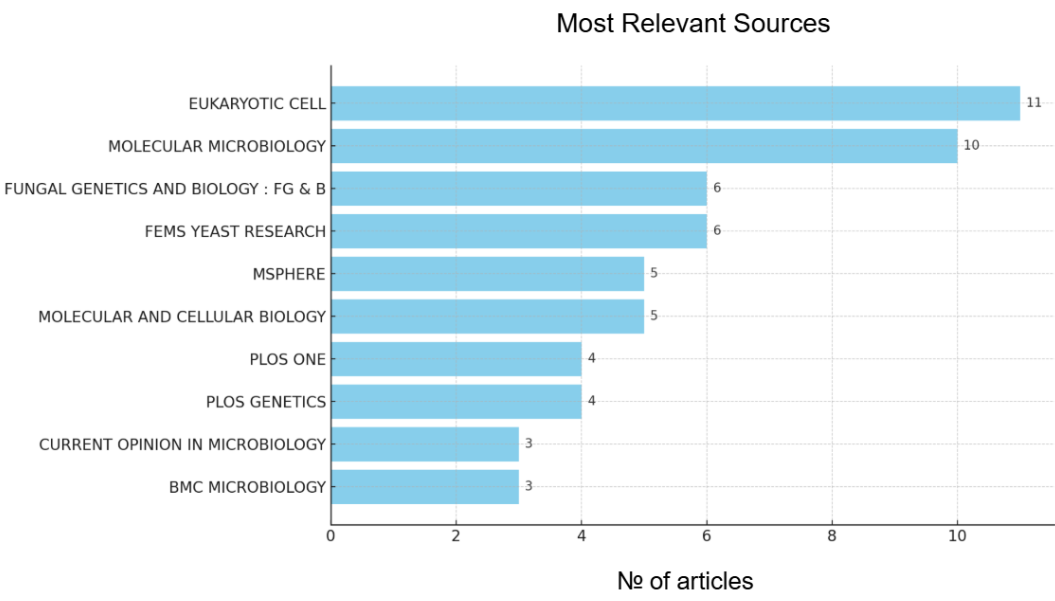
Figure 4. Journals ranked by publications' frequency related to fungal kinase through scientometric analysis with the Bibliometrix R-package based on PubMed database (~270 articles) between 1982 and 2025. These journals are principal platforms of research dissemination in fungal pathogenesis, signal transduction, and kinase biology.


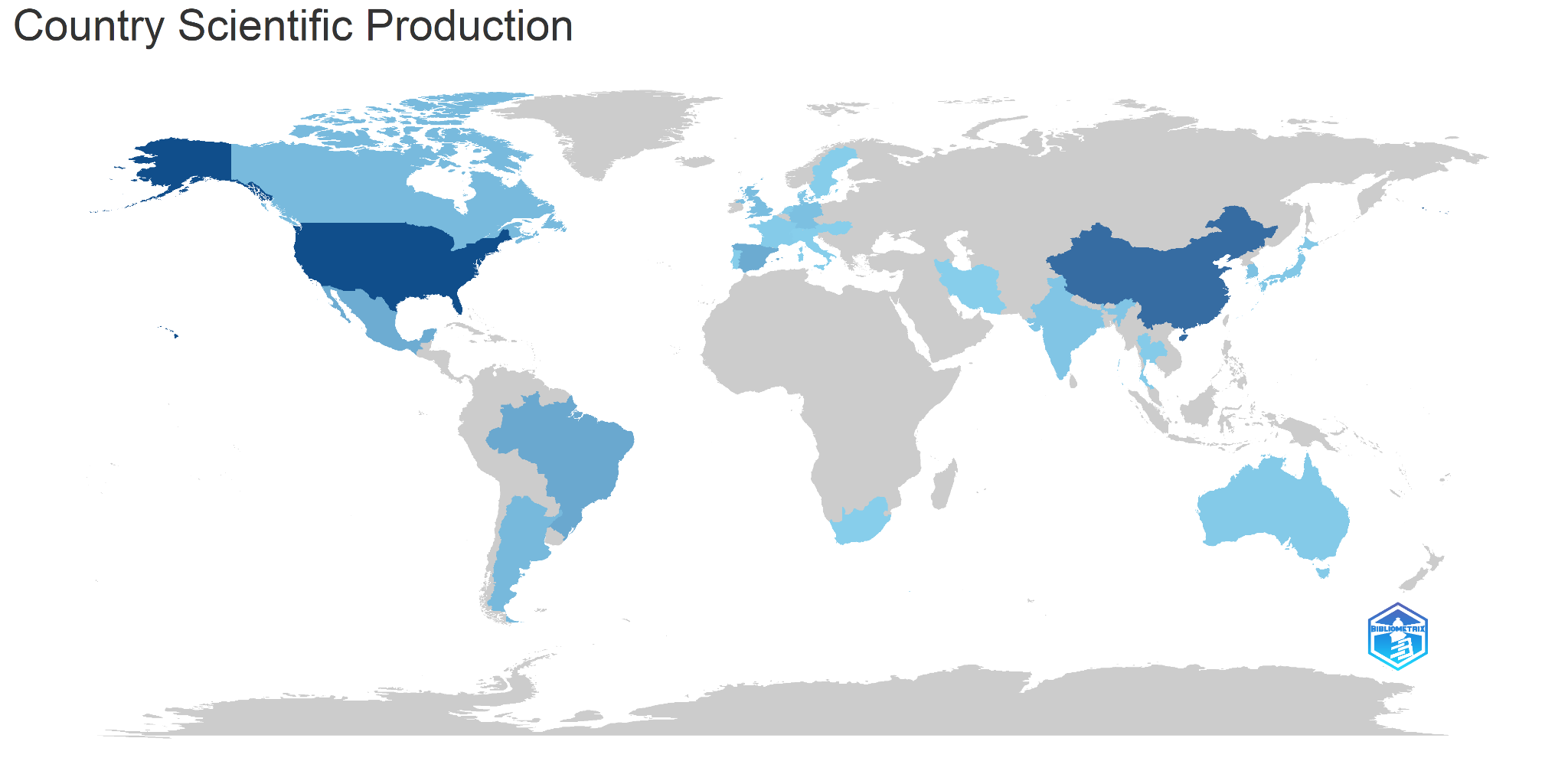


Figure 5. Geographic distribution of scientific output related to dimorphic fungal kinases. The map demonstrates countries contribution on dimorphic fungi kinases research, based on PubMed database (~270 articles) between 1982 and 2025 and analyzed with the Bibliometrix R-package. Darker shades indicate higher publications' number.


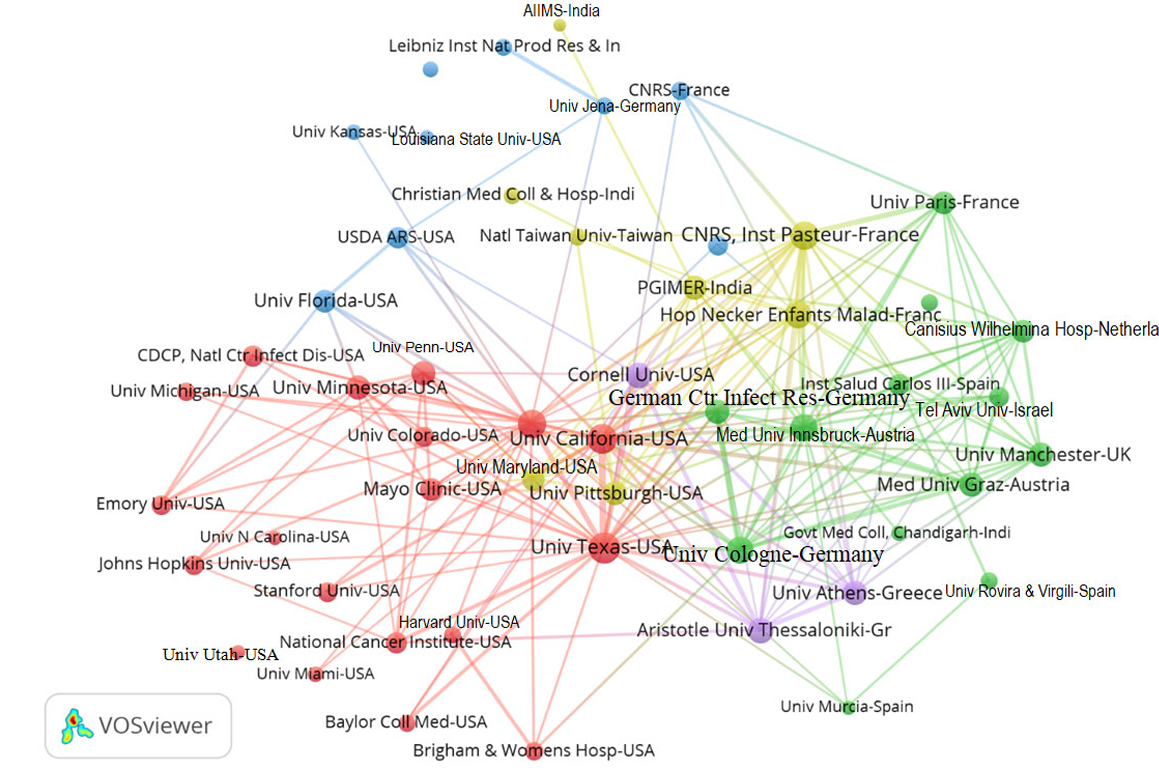


Figure 6. Network institutional collaborations related to dimorphic fungal kinases, generated using VOSviewer based on PubMed database (~270 articles) between 1982 and 2025. Each circle represents a research institution and its size is proportional to publications' volume. Lines demonstrates co-author relationships.
